# Functional and Hybrid Imaging of Bone Metastases

**DOI:** 10.1002/jbmr.3444

**Published:** 2018-05-23

**Authors:** Gary JR Cook, Vicky Goh

**Affiliations:** 1 Department of Cancer Imaging, School of Biomedical Engineering and Imaging Sciences, King’s College London, St Thomas’ Hospital, London SE1 7EH, United Kingdom; 2 King’s College London and Guy’s & St Thomas’ PET Centre, St Thomas’ Hospital, London SE1 7EH, United Kingdom; 3 Radiology Department, Guy’s & St Thomas’ Hospitals, London SE1 7EH, United Kingdom

**Keywords:** Bone metastases, Single photon emission computed tomography, Positron emission tomography, Whole-body diffusion-weighted magnetic resonance imaging

## Abstract

Bone metastases are common, cause significant morbidity, and impact on healthcare resources. Although radiography, computed tomography (CT), magnetic resonance imaging (MRI), and bone scintigraphy have frequently been used for staging the skeleton, these methods are insensitive and nonspecific for monitoring treatment response in a clinically relevant time frame. We summarize several recent reports on new functional and hybrid imaging methods including single photon emission CT/CT, positron emission tomography/CT, and whole-body MRI with diffusion-weighted imaging. These modalities generally show improvements in diagnostic accuracy for staging and response assessment over standard imaging methods, with the ability to quantify biological processes related to the bone microenvironment as well as tumor cells. As some of these methods are now being adopted into routine clinical practice and clinical trials, further evaluation with comparative studies is required to guide optimal and cost-effective clinical management of patients with skeletal metastases.

## Introduction

Bone metastases are common, particularly in patients with two of the most common cancers, breast and prostate cancer, where up to 70% of patients are affected.^(1)^ Skeletal-related events secondary to bone metastases from any cancer are associated with significant morbidity such as pain, hypercalcemia, fractures, bone marrow suppression, and spinal cord compression.^(2)^ With more effective, but more costly, therapeutics for metastatic breast and prostate cancer, survival is relatively long compared to other cancers, and so healthcare costs are high.^(3,4)^ It is, therefore, not only important to diagnose skeletal metastases as early as possible, but to determine which patients are not responding to therapy. An early transition to second-line therapy can then be considered with the aims of reducing toxicity from ineffective treatment and increasing quality of life and progression-free and overall survival.

There is increasing use of biochemical markers of bone turnover and tumor-derived markers in the diagnosis and monitoring of skeletal metastases, but these are less able to determine overall skeletal burden than imaging methods and are unable to localize sites of disease or predict complications.^(1)^ Nevertheless, these biomarkers have a complementary role in imaging in the management of patients with skeletal metastases.

Although bone scintigraphy has traditionally been used for detecting skeletal metastases and monitoring therapy, it is recognized that sensitivity and specificity are limited, both in detection and for monitoring treatment response. Conventional imaging, such as radiography, computed tomography (CT), or magnetic resonance imaging (MRI), that relies on size-based criteria for assessing treatment response (eg, Response Evaluation Criteria in Solid Tumors [RECIST]^(5)^), is also limited as bone disease is usually considered non-measurable unless associated with a measurable soft tissue component. Attempts have been made to incorporate bone scintigraphy with other imaging in breast cancer^(6)^ and prostate cancer^(7)^ to improve response assessment, but early assessment within a clinically relevant time frame remains problematic in clinical practice.

The combination of either tumor-or bone-specific radio-tracers with CT or MRI in hybrid scanners, such as single photon emission computed tomography (SPECT/CT), positron emission tomography/CT (PET/CT), or PET/MRI, have the potential to improve diagnosis and response assessment with synergy between morphological and molecular information. However, despite the potential for gathering multiparametric information from metastases that reports on diverse underlying biological and morphological tumor characteristics, there have been relatively few reports that have successfully exploited these potential benefits.

The purpose of this review is to provide an update on the current status of functional and hybrid imaging, particularly PET and functional MRI methods, in detection and therapy-response monitoring of bone metastases with discussion of some potential future methods that show promise (Table 1).

## Pathophysiology Relevant to Imaging

Paget’s proposal, that metastases are the result of an interaction between the seeds (cancer cells) and soil (organ microenvironment), is relevant to skeletal metastasis and imaging.^(8,9)^ Tumor-specific imaging agents may be able to detect metastatic disease at an early stage while within the bone marrow (soil) compared to bone-specific imaging methods that require a subsequent change in the bone itself. Therefore, bone marrow imaging, such as MRI or tumor-specific imaging (eg, diffusion-weighted MRI or 18F-fluorodeoxyglucose [18F-FDG] PET) may detect skeletal metastases before imaging methods that rely on changes in mineralized bone tissue (eg, radiography, CT, bone scintigraphy, 18F-fluoride PET).

Morphological characteristics of untreated bone metastases vary on a spectrum between lysis (osteolytic) and sclerosis (osteoblastic) and reflect different underlying biological mechanisms. Although lytic metastases are more common and typically occur in lung and breast cancer, sclerotic metastases are usually seen in prostate cancer. Whereas either osteolytic or osteoblastic processes may predominate in a particular metastasis or tumor type, there is usually a mixture of both processes to some extent.

In the commoner type of metastasis that is predominantly osteolytic, factors, such as parathyroid hormone-related protein (PTHrP) derived from cancer cells, stimulate osteoblast production of the receptor activator of the nuclear factor-κB ligand (RANKL), which in turn stimulates osteoclast maturation and activity.^(10)^ The increased osteoclast activity leads to increased local bone resorption at a greater rate than attempts at osteoblastic bone formation and repair, with net loss of bone. With the subsequent release of growth factors from the bone matrix, such as TGF-β, there is further stimulation of PTHrP and hence a resultant vicious cycle of bone destruction. In metastases where osteoblastic processes predominate, a number of tumor-derived growth factors (eg, platelet-derived growth factor) contribute to this phenotype by stimulating osteoblasts. It is recognized that there is increased osteoblastic activity in lytic metastases and osteoclastic activity in metastases that are predominantly sclerotic.^(11)^ During healing following successful treatment, both lytic and sclerotic metastases become more sclerotic and osteoblastic bone formation and repair occurs.^(12,13)^

Functional- and molecular-imaging agents used for the detection of skeletal metastases can be broadly divided into bone- and tumor-specific agents. Bone-specific agents such as 99m-technetium-labeled methylene diphosphonate and 18F-fluoride have been used as a SPECT tracer (bone scan) and a PET tracer, respectively. These agents have similar uptake mechanisms and depend, to some extent, on local blood flow, but mainly on osteoblastic mineralization activity, whereby the labeled molecule is incorporated into mineralizing bone.^(14)^ Despite these agents showing higher accumulation in osteo-blastic metastases, they are sensitive methods for the detection and staging of most cancers that are predominantly osteolytic, such as lung and breast cancer, but relatively insensitive in purely osteolytic disease such as myeloma.^(15,16)^ A disadvantage of these imaging agents is that they cannot differentiate osteoblastic activity caused by tumor progression and growth from that which occurs following successful therapy. As such, an increase in activity, or indeed the appearance of new previously inconspicuous lesions, can be seen at metastatic sites for several weeks: the so-called flare phenomenon.^(17–19)^ An area of academic interest that has not yet reached the clinic is the imaging of the osteoclast activity associated with skeletal metastases. There has been interest in radiolabeling osteocalcin to exploit the receptors for this peptide on osteoclasts.^(20)^ Recently, there has been interest as well in radiolabeled compounds that contain the asparginine-glycine-aspartate (RGD) motif that binds strongly to integrins such as α_v_β_3_. Osteoclasts express more α_v_β_3_ integrin than any other cell, adhering to bone matrix via this integrin during bone resorption.^(21)^ Preclinical experiments in osteolytic metastatic and PTHrP-induced calvarial models have demonstrated osteo-clast-specific accumulation.^(22,23)^ In man, radiolabeled RGD compounds have shown accumulation in bone metastases from lung cancer and also prostate cancer.^(24,25)^ In the latter report,^(25)^ an inverse correlation was seen between lesion uptake and CT density in keeping with an osteoclastic mechanism of uptake. A reduction in activity was also noted in patients who responded to systemic treatment with abiraterone compared to those who had progressive disease.

Tumor-specific imaging methods rely on different underlying cellular biological characteristics of tumors for contrast in the image. Conventional MRI (T1, T2, short T1-inversion recovery) detects differences in proton density (water content) in tumors compared to normal bone marrow; the signal from diffusion-weighted MRI relates to the restriction of water-molecule motion and can be quantified by measurement of the apparent diffusion coefficient (ADC).^(26,27)^ Highly cellular tumors show greater restriction of water-molecule motion than normal bone marrow. Examples of PET tumor-specific tracers that show uptake in skeletal metastases include 18F-FDG (cellular glycolysis), 18F-choline (cellular choline kinase activity and membrane turnover), and 68Ga-prostate-specific membrane antigen (68Ga-PSMA; cellular PSMA expression in prostate and some other cancers).^(28–30)^

## X-Ray-Based Imaging Methods

Radiography and CT demonstrate the morphological consequences of metastases that change the density of bone secondary to local changes in mineralization, as a result of osteolytic or osteoblastic activity. The poor sensitivity of radiographs, requiring up to 50% of bone to be destroyed before lytic metastases are visible,^(31)^ and slow or absent changes following successful therapy, are well-recognized.^(32)^ Similar to bone scintigraphy, CT can show an osteoblastic flare in healing metastases following successful treatment, either by showing an increase in density and sclerosis or the appearance of new, previously occult lesions.^(13)^

## Bone Scintigraphy Including SPECT and SPECT/CT

**Fig. 1:**
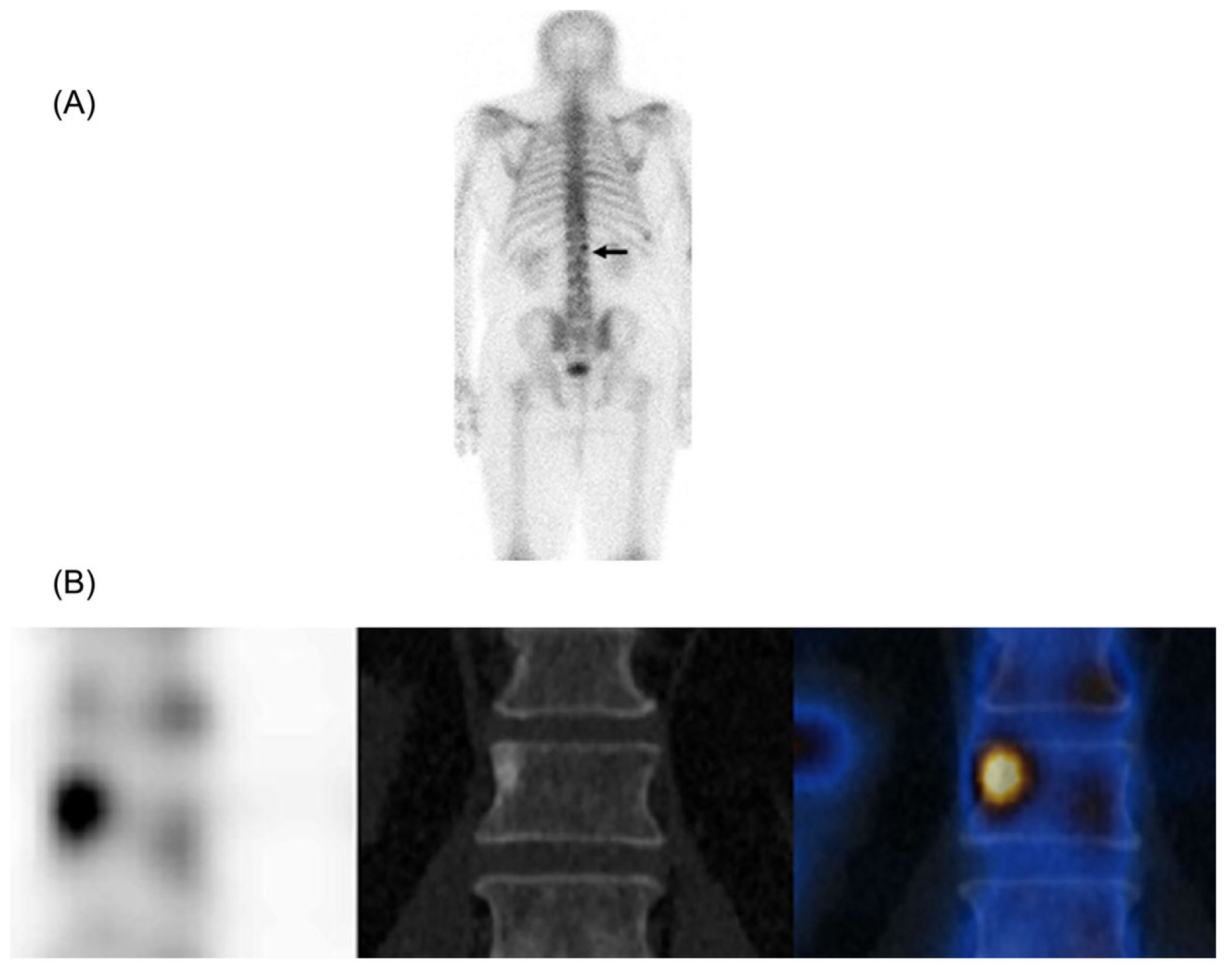
A man with a new diagnosis of high-risk prostate cancer and elevated prostate-specific antigen. The posterior planar scan (*A*) shows a small focus of activity at L1 which is difficult to characterize. The coronal single-photon-emission computed tomography (SPECT)/CT images (*B*) show higher contrast resolution on SPECT (left), a typical sclerotic focus on CT (middle) combined on the fused SPECT/CT image (right) with the typical appearance of a metastasis.

For several decades bone scintigraphy has been the standard method for staging the skeleton in most cancers and for monitoring treatment response, although it is now accepted that there are limitations in specificity and sensitivity in detecting disease and in specificity in monitoring treatment response.^(32,33)^ The addition of tomographic scan acquisitions (SPECT), followed by the availability of hybrid SPECT/CT gamma cameras, has helped improve both sensitivity and specificity in diagnosing skeletal metastases.^(34–36)^ The greatest improvement has been in specificity, where the CT component of the scan has allowed each scintigraphic hot spot to be more accurately categorized as benign or malignant by including the morphological appearances, leading to an increase in confidence in reporting scans with fewer equivocal studies.^(36)^ An increase in sensitivity is also reported, resulting from the increased contrast resolution available with SPECT compared to standard planar imaging (Fig. 1*A* and 1*B*).

As discussed above, a disadvantage of bone scintigraphy is an inability to differentiate an increase in uptake (or new lesions) caused by the flare phenomenon from progressive disease for several weeks or months following the commencement of new systemic endocrine therapy or chemotherapy.^(17–19)^ If a flare is present, it is a favorable prognostic sign.^(19)^ The flare can also be used to improve diagnostic accuracy. For example, in high-risk prostate cancer patients at initial staging, when bone scintigraphy was repeated 6 weeks after commencing endocrine treatment, a flare occurred in 9 of 22 (41%) patients who had unequivocal bone metastases, 4 of 36 (11%) patients with negative scans became positive for bone metastases, and a flare occurred in 8 of 41 equivocal baseline scans, a sign that was 100% specific.^(37)^

The addition of SPECT or SPECT/CT probably does not improve the performance of bone scintigraphy for response evaluation in clinical practice or as an end point in clinical trials. Nevertheless, it is accepted that SPECT and SPECT/CT show additional benefit in staging the skeleton compared to planar bone scintigraphy alone.

## PET and PET/CT

### 18F-fluoride

**Fig. 2:**
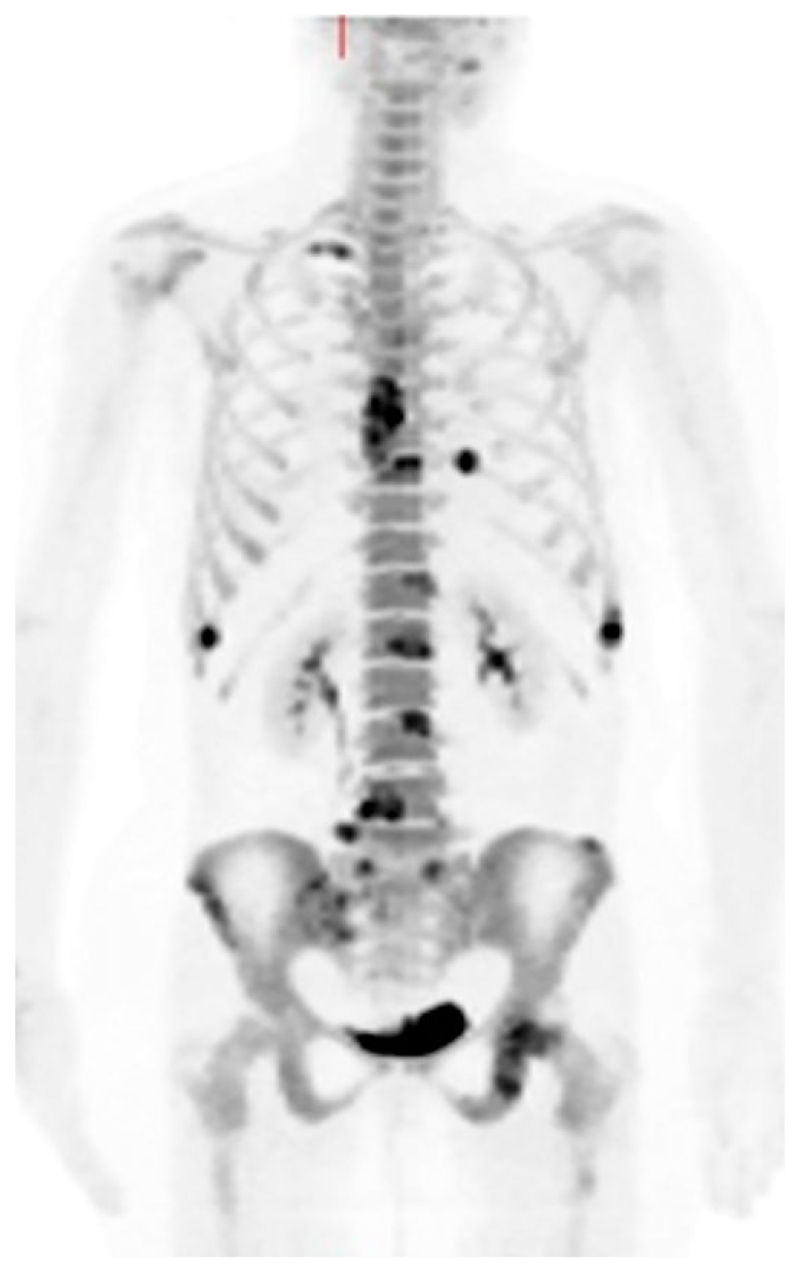
A woman with metastatic breast cancer. An 18F-fluoride positron emission tomography maximum-intensity projection image shows high tracer uptake in several metastatic lesions.

18F-fluoride is a bone-specific PET tracer that was first described in 1962 before the more ubiquitous use of 99mTc-labeled diphosphonate agents for imaging with gamma cameras.^(38)^ Uptake depends on local blood flow and active mineralization, where the fluoride ions replace hydroxyl ions in hydroxyapatite to form fluoroapatite in bone mineral. Skeletal uptake, with near 100% first-pass extraction by bone and background clearance by renal excretion, is more rapid than with 99mTc-labeled bone agents; images can be acquired within 60 minutes of injection. These properties, combined with the superior spatial and contrast resolution of PET compared to gamma camera scintigraphy and SPECT, allow high-quality functional images of the skeleton (Fig. 2).^(14)^

Absolute quantification is possible with PET; therefore, there has been interest in quantitative imaging of the skeleton with the ability to estimate lesional or regional blood flow and mineralization activity (plasma clearance of 18F-fluoride to the bone mineral compartment), as well as other physiological parameters.^(14,16)^ Good correlations have been shown with skeletal histomorphometry, allowing a noninvasive measurement of regional skeletal metabolism.^(39,40)^ Most of the published literature on quantitative 18F-fluoride PET kinetics has concentrated on benign skeletal disease, but some work exists on using kinetic 18F-fluoride PET parameters to monitor treatment response in skeletal metastases.^(41–43)^ The disadvantages of quantitative PET for measuring kinetic parameters are that only a relatively small part of the skeleton can be included (approximately 10 to 20 cm *z* axis) and that a dynamic scan of approximately 60 minutes, as well as arterial blood sampling, are required. However, methods have been introduced to simplify this methodology to obtain noninvasive arterial input functions from image or population data and to estimate kinetic parameters from static scans of the whole skeleton.^(44–46)^

Despite the quantitative advantages of PET, most of the published literature describing either the staging or response assessment of skeletal metastases has been qualitative or semiquantitative. However, 18F-fluoride PET and PET/CT studies in breast, prostate, lung, and other cancers have shown improved diagnostic accuracy compared to bone scintigraphy +/- SPECT or CT.^(47–53)^ The impact of 18F-fluoride PET/CT on the management of patients with cancers other than prostate cancer was assessed in a National Oncology PET Registry (NOPR) trial that included 1814 patients (781 breast, 380 lung, and 653 other cancers).^(54)^ For suspected first osseous metastasis, 18F-fluoride PET/CT led to management changes in 24%, 36%, and 31% of patients with breast, lung, and other cancers, respectively. In patients with suspected progressive osseous disease, management changed in 60% of breast cancer and 52% of other cancers (lung cancer not recorded). In a similar study of 3531 patients with prostate cancer (1024 initial staging, 1997 first osseous metastasis, 510 progressive osseous disease), change in management from nontreatment to treatment occurred in 47%, 44%, and 52%, respectively.^(55)^

The measurement of total skeletal metastatic burden is possible with 18F-fluoride PET as there is generally high contrast between metastases and normal bone. In prostate cancer, a number of studies have shown that global quantitative metrics can predict treatment response and progression-free survival or overall survival.^(56,57)^

There are fewer published data on the use of 18F-fluoride PET/CT to monitor treatment response in skeletal metastases. Early studies have shown potential utility in monitoring early treatment response at 12 weeks to 223Ra-radium chloride^(58)^ and dasatinib.^(42)^ With respect to 223Ra-radium chloride treatment in metastatic prostate cancer, 18F-fluoride PET can predict absorbed dose to metastases^(59)^ and risk of bone marrow toxicity.^(60)^ In another NOPR study of 2217 patients evaluating the efficacy of using 18F-fluoride PET/CT to monitor treatment response, predominantly with prostate (68%), breast (17%), and lung cancer (6%), an overall change in management was found in 40%.^(61)^ In breast cancer patients on endocrine treatment, 18F-fluoride PET/CT may show heterogeneity of response within and between patients, an observation that can be partly explained by the flare phenomenon.^(62)^

### 18F-Fluorodeoxyglucose

18F-Fluorodeoxyglucose is regarded as a tumor-specific PET tracer relying on the Warburg effect of increased glucose transporters and glycolysis by hexokinase II in most malignant tumors for contrast between tumor and normal cells.^(63)^ Interestingly, different skeletal metastatic phenotypes appear to show different avidity to 18F-FDG. Osteoblastic metastases characteristically show low or absent uptake, whereas osteolytic lesions tend to be more 18F-FDG-avid and to be associated with a worse prognosis.^(64–66)^ This phenomenon is most recognized in breast cancer; the low avidity of sclerotic metastases seems to occur both in treated and untreated disease, especially in the lobular cancer subtype.^(67)^ In previously treated disease, despite 18F-FDG-negative appearances caused by nonviable tumor cells, increased osteoblastic activity demonstrated by increased activity on bone scintigraphy or 18F-fluoride PET, may persist much longer.^(68)^ Prostate cancer, typically associated with osteoblastic metastases, also tends to show low 18F-FDG avidity in bone and soft tissue metastases, and so other PET tracers are preferable for detecting metastatic disease (see below).

In most cancers, osteolytic disease predominates and there are several reports, including meta-analyses, of 18F-FDG PET/CT showing greater diagnostic accuracy than bone scintigraphy in most cancers, but particularly in breast cancer.^(64,69–72)^

**Fig. 3:**
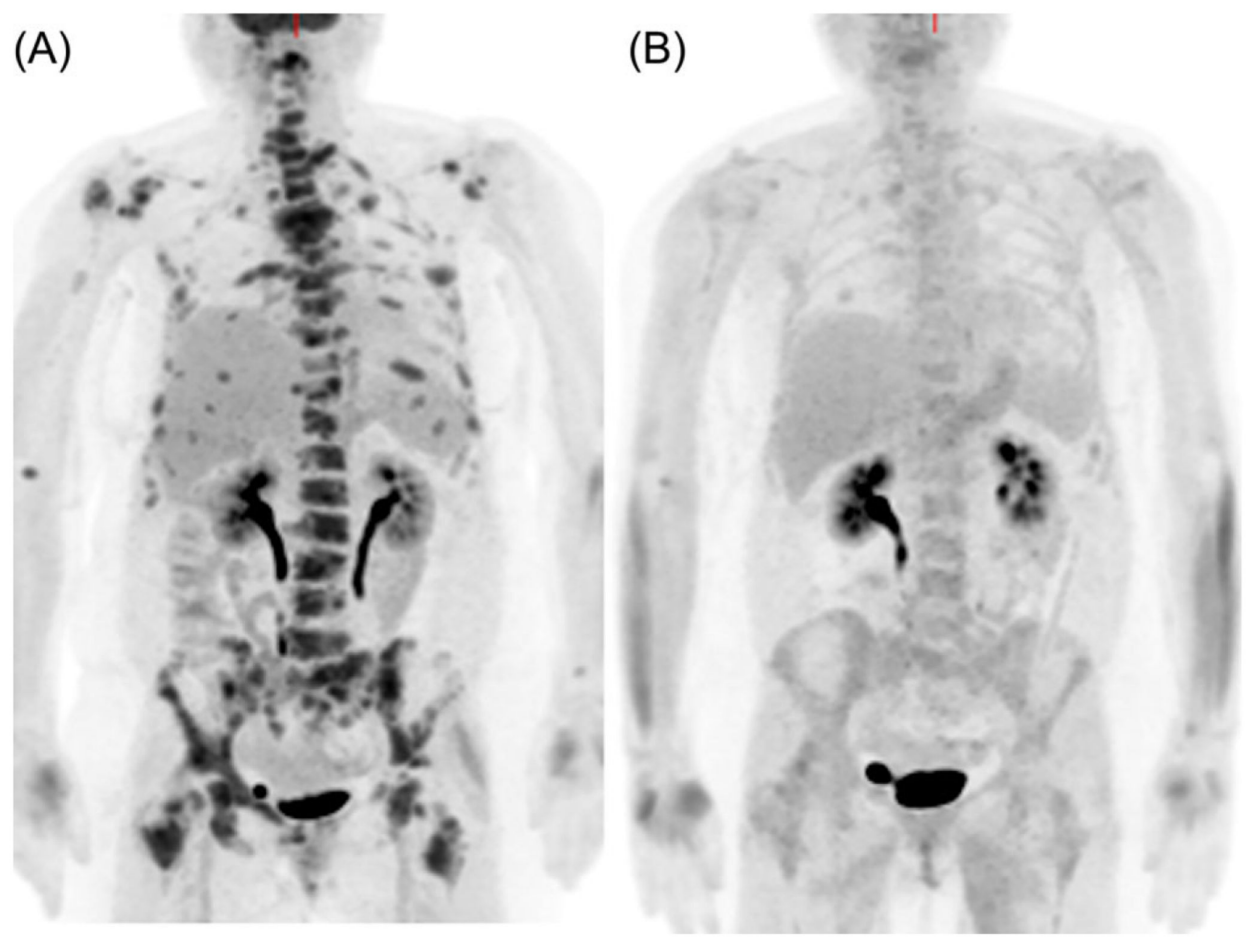
A woman with metastatic breast cancer. Fluorodeoxyglucose-positron emission tomography maximum-intensity projection images before (*A*) and 8 weeks (*B*) after commencing endocrine treatment show a metabolic response at all skeletal metastatic sites.

A weakness of conventional imaging is an inability to accurately measure early treatment response or nonresponse, an area where it is hoped that functional imaging can improve clinical practice. It is in this area that there is accumulating evidence that 18F-FDG PET/CT is clinically useful; it has entered routine practice in some institutions. Taking advantage of the metabolic information to interpret the morphological changes is possible with combined PET/CT and increases specificity. It has been reported that progressive breast cancer bone metastases become more lytic and 18F-FDG-avid, but increased sclerosis can be associated with response and progression.^(73,74)^ Whereas reduction in 18F-FDG uptake in metastases (as measured by the maximum standardized uptake value [SUVmax]) and increased sclerosis on CT has been reported to predict time to progression, only SUVmax remained significant in a multivariate model.^(75)^ Additional studies have shown associations between changes in 18F-FDG uptake and tumor markers, circulating tumor cells, time to progression and skeletal-related events, following systemic endocrine and chemotherapeutic regimes (Fig 3*A* and 3*B*).^(76–79)^ Unlike 18F-fluoride PET or bone scintigraphy, metabolic flare is not a commonly recognized phenomenon in 18F-FDG PET imaging of skeletal metastases and does not commonly cause difficulties in differentiating progressive disease from posttherapy healing in clinical practice. However, a flare has been reported in a small number of patients with lung cancer treated with the antiangiogenic agent bevacizumab in combination with standard chemotherapy.^(80)^ If there is a discordant response between metastases in an individual patient, this probably reflects true intermetastatic heterogeneity of response, an increasingly recognized phenomenon resulting from the polyclonal differentiation of cancer.^(62,81)^

### Other PET tracers

The high prevalence of bone metastases, combined with the low uptake of the most commonly used PET tracer 18F-FDG, in prostate cancer has led to alternative tracers being used in this cancer. The osteoblastic nature of prostate cancer bone metastases means that 18F-fluoride PET is very sensitive and, when combined with CT, quite specific in detecting skeletal disease. However, with the potential problems from the flare phenomenon and relatively little published data, the use in monitoring treatment response is currently limited, despite promising results from the NOPR study.^(61)^

There has therefore been interest in a number of tumor-specific tracers relating to tumor metabolism and antigen expression. Choline, labeled either with 11C-carbon or 18F-fluorine, has become a standard clinical tracer for staging high-risk prostate cancer and patients with biochemical recurrence. Uptake is seen in osteoblastic metastases, with even higher activity noted in the rarer osteolytic phenotype.^(82)^ In patients treated with hormones, the most sclerotic lesions on CT were noted to be choline-negative despite continued 18F-fluoride activity, an observation interpreted as showing a lack of tumor cell viability posttreatment, but with continued osteoblastic healing.^(83)^

At initial staging of high-risk prostate cancer, choline PET/CT has shown higher accuracy than bone scintigraphy in a number of studies^(84–86)^ with similar results in those with biochemical recurrence.^(87)^ In a further study that compared 18F-choline and 18F-fluoride PET/CT in patients with prostate cancer, some patients showed metastases with 18F-choline, but not 18F-fluoride (interpreted as showing small-volume bone marrow deposits before sufficient osteoblastic activity to be visible with 18F-fluoride) and vice-versa.^(88)^ As yet, there are no strong prospective data to support the use of choline PET/CT in monitoring treatment response. Changes in 11C-choline uptake after docetaxel chemotherapy were reported as valuable in identifying patients with progressive disease despite apparent prostate-specific antigen (PSA) response.^(89)^ In monitoring response to novel endocrine therapies, results are conflicting. One study evaluating enzalutamide response reported that baseline SUVmax of 18F-choline PET predicted survival^(90)^; however, another described no additional value over the measurement of PSA alone.^(91)^ In contrast, early imaging at 3 and 6 weeks is predictive of outcome better than PSA response in castrate-resistant disease treated with abiraterone.^(92)^

**Fig. 4:**
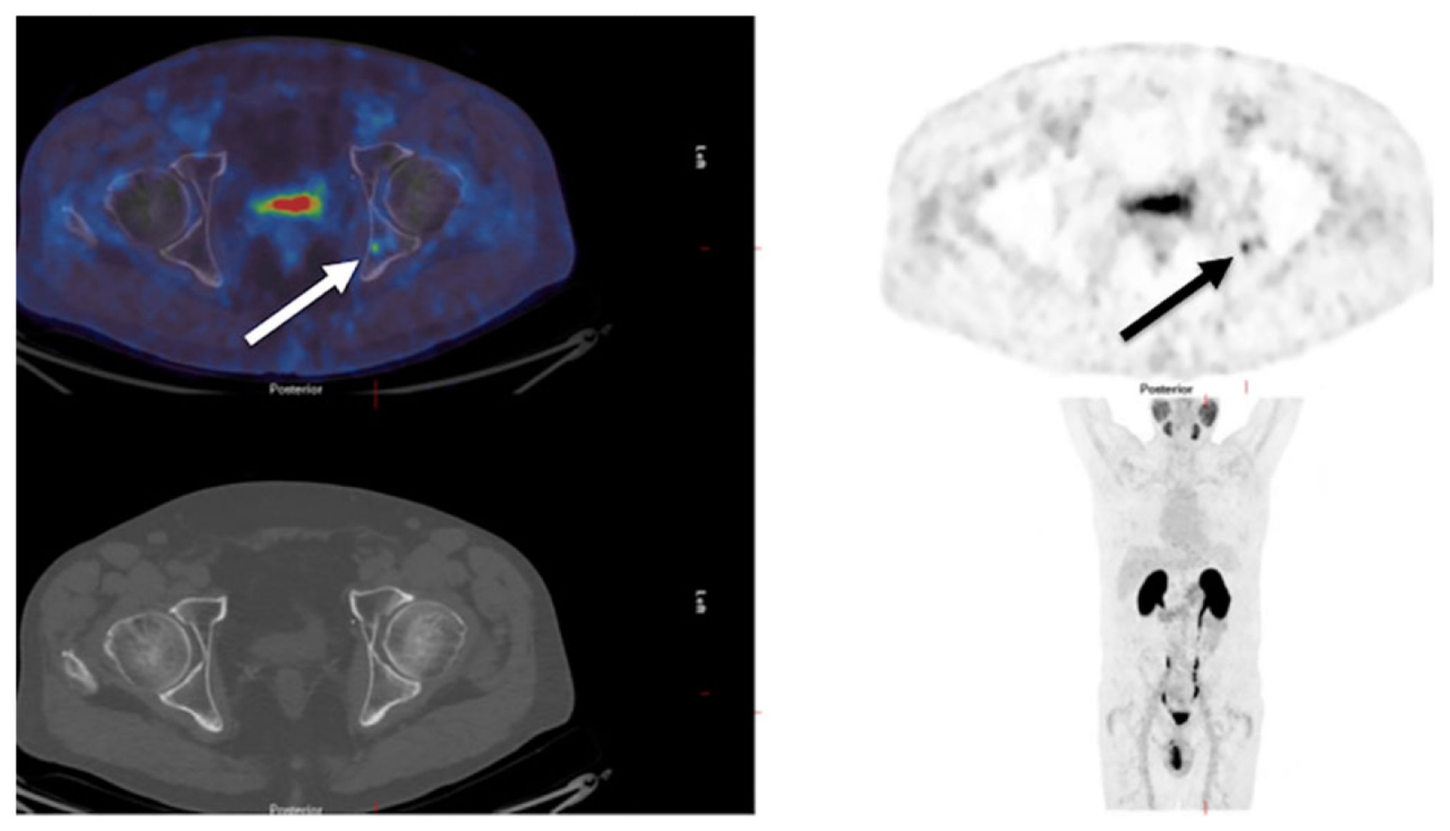
A man with biochemical recurrence of prostate cancer. A 68Ga-prostate-specific membrane antigen positron emission tomography/computed tomography (PET/CT) scan shows a small metastasis in the left posterior acetabulum (arrows), invisible on the CT component of the study (bottom left).

68Ga-Prostate-specific membrane antigen tracers have been a recent introduction to clinical practice with advantages in sensitivity, specificity, and tumor-to-background contrast (particularly in the skeleton) in comparison to 18F-choline PET/CT (Fig. 4).^(93)^ Although the level of evidence currently remains low, 68Ga-PSMA is superior to bone scintigraphy in primary staging; however, evidence in biochemical recurrence is still awaited.^(94–96)^ The superior sensitivity of PSMA-based imaging reflects the high expression of this antigen in prostate cancer cells as it is a folate hydrolase that is implicated in cellular folate uptake and proliferation.^(97)^

### Magnetic resonance imaging

Although conventional spin-echo-based MRI sequences return a poor signal from mineralized bone, these sequences are sensitive to bone marrow and to tumors within bone marrow; therefore, they may detect metastases before significant bone destruction or sclerosis has occurred.^(98)^ The sensitivity of conventional MRI has been increased with the development and clinical integration of new MRI sequences. These include spin-echo T2-weighted^(99)^ and gradient-echo T1-weighted sequences with Dixon fat suppression, which produce fat- and water-dominant images as well as allow the measurement of skeletal fat fraction.^(100)^ In addition, diffusion-weighted sequences, which assess proton diffusion, produce images with increasing b-weighting and allow the ADC to be measured.^(101)^ Metastases appear of lower signal on fat-dominant images and of higher signal on water-dominant images. Metastases typically appear of higher signal on increasing b-value diffusion sequences and of higher ADC than the normal bone marrow related to the difference in cell size and distribution compared to normal fat cells.

### Whole-body MRI

Whole-body MRI (WB-MRI) acquisitions are now feasible in times of less than 1 hour, usually 30 to 60 minutes, for the detection and characterization of skeletal lesions.^(102)^ In addition to the standard morphological sequences, including T1-weighted and T2-weighted sequences, diffusion-weighted imaging (DWI) is now frequently included.^(26,27,103)^ The lack of ionizing radiation, high spatial- and tissue-contrast resolution, and high sensitivity are advantages of WB-MRI such that WB-MRI is being adopted into clinical protocols with recent guidance on acquisition and interpretation having been published for metastases from prostate cancer.^(100)^

The potential disadvantages of WB-MRI include motion artifacts during the relatively long acquisition time, limited access to busy MRI scanners in many institutions, and the possibility that the addition of DWI, while improving sensitivity, may reduce specificity.^(103)^ Nevertheless, several reports, showing high diagnostic accuracy in breast and prostate cancer, demonstrate comparable results using 18F-choline, 18F-fluoride, and 18F-FDG PET/CT.^(104–107)^

### Diffusion-weighted and dynamic contrast-enhanced MRI

**Fig. 5:**
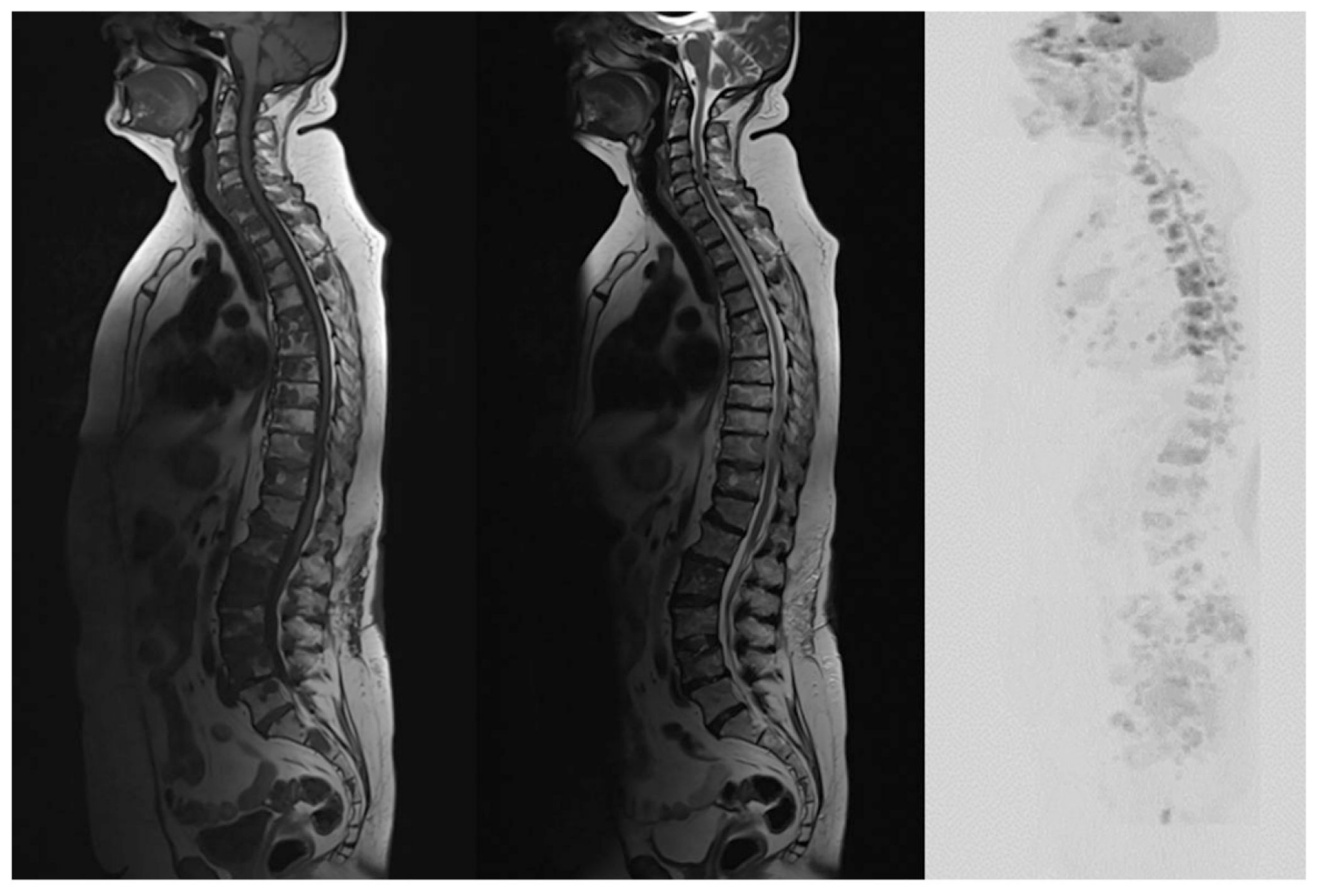
T1-weighted (left), T2-weighted (center), and diffusion-weighted inverted maximum-intensity projection (b800 s/mm^2^) (right) sagittal sequences demonstrating multiple bone metastases in a patient with metastatic breast cancer. The metastases show a low signal on T1-weighted images, a low-to-intermediate signal on T2-weighted images, and an increased signal on diffusion-weighted imaging.

The biophysical basis of DWI is the microscopic displacement of water molecules as a result of thermal Brownian motion. In cancer, the tumor environment restricts this motion (Fig. 5). This can be quantified by the ADC, which reflects the rate of signal loss with increasing b-weighting applied and is a measurement of the effective displacement of water molecules.^(27,108)^ Tumor ADC from b-values of <1000 s/mm^2^ is a surrogate of the extracellular space, although cell size, cell arrangements, cell density, integrity of cell membranes, glandular structures, and extracellular space viscosity and tortuosity will influence this—hence why reduced ADC has been attributed to higher cell density. The ADC typically increases with successful therapy when water molecules are more freely diffusible within the extracellular space as a result of cytotoxicity and reduced cell membrane integrity.

There has been great interest in the use of DWI to provide a quantitative measure of treatment response in skeletal metastases by measuring an increase in the diffusion of water molecules that result from lower cellularity following successful treatment.^(108,109)^ Early reports suggest efficacy in determining early response in prostate cancer.^(110,111)^ However, a challenge with sclerotic bone lesions is that there are fewer protons to produce a signal; thus sclerotic metastases will return a low signal on T1- and T2-weighted MRI. They are also associated with lower diffusion and low ADC. Therefore, a limitation is differentiating sclerosis following successful treatment from progressive disease,^(110)^ similar to 18F-fluoride PET and bone scintigraphy. Nevertheless, this has not been shown to have a significant negative diagnostic effect in prostate cancer.^(112)^

Dynamic contrast-enhanced (DCE) MRI refers to the rapid acquisition of a time series of T1-weighted images before, during, and after intravenous administration of a gadolinium-based contrast agent. Gadolinium contrast agents are small hydrophilic molecules with a short circulation half-life, typically <1 hour. These contrast agents shorten the T1-relaxation rate, thus causing signal enhancement related to the delivery and leakage rate of the contrast agent within the tissue of interest, providing a surrogate measure of angiogenesis. From kinetic modeling, the rate of perfusion and vascular leakage can be measured (transfer constant, K^trans^) and the rate of return into the vascular system (rate constant, k_ep_), as well as the fractional extravascular extracellular volume (v_e_) and plasma volume (v_p_).^(113)^

The presence of disseminated tumor cells in bone marrow in breast cancer patients may shift K^trans^ and k_ep_ toward lower values.^(114)^ Lesional K^trans^ values may also differ depending on mutational status: Higher K^trans^ values are noted in epidermal growth factor receptor mutated non-small cell lung cancer bone metastases.^(115)^ Changes in qualitative or quantitative parameters following therapy have been shown in animal models^(116,117)^ and in patients with breast cancer bone metastases (change in T1 curve shape^(118)^) and other bone metastases (reduction in K^trans^).^(119)^ A reduction in plasma volume (vp) in spinal metastases is a good prognostic indicator after radiotherapy.^(120)^

### Future directions

With the increased availability of functional and hybrid imaging, many of the methods described above are accessible in the clinic. Although these methods offer improved diagnostic accuracy, it is unclear which method works best in each cancer type and at what stage of the management pathway. Comparative studies are required, preferably multicenter ones that will help standardize protocols and analysis. The best analysis method for skeletal metastases is still undetermined: Should a sample of metastases be selected with the risk of not taking into account lesion heterogeneity, or should all metastases be included in a whole-body-skeletal burden method? The clinical impact of heterogeneity of response between metastases in an individual patient also needs to be evaluated. In addition to refining current techniques, targeting other aspects of abnormal metastasis or bone microenvironment biology such as osteoclasts may be fruitful, given their central role in most skeletal metastases and the number of treatments aimed at osteoclast activity that are reaching the clinic.

## Conclusions

There is no doubt that functional and hybrid imaging methods including SPECT/CT, PET/CT, and WB-MRI complemented with DWI and PET/MRI can improve the detection of skeletal metastases; data suggest that an earlier and more accurate treatment response is possible. Some of these methods, including 18F-FDG PET/CT and WB-MRI with DWI are already entering routine clinical practice; however, large-scale studies with health economics analyses are required to guide our best practices and our optimal clinical management of patients with skeletal metastases.

## Figures and Tables

**Table 1:** Summary of the Main Characteristics of Functional and Hybrid Imaging of Bone Metastases

Modality	Hybrid modality	Ionizing radiation	Subtype	Mechanism	Advantages	Disadvantages
Radiography		Yes		Calcium in mineralized bone causes contrast in image	Inexpensive, widely available, relatively low radiation dose, high spatial resolution	Insensitive for detection and response assessment, morphology only, low contrast resolution
CT	SPECT/CT, PET/CT	Yes		Calcium in mineralized bone causes contrast in image.	Widely available, high contrast resolution, tomographic images in any plane, also reports on soft tissue disease	Insensitive for detection and response assessment in bone; morphology only
Bone scintigraphy	SPECT/CT	Yes	99mTc-MDP	Uptake depends on blood flow and mineralization rate.	Widely available, inexpensive, sensitive for detection, high contrast resolution (augmented by SPECT)	Uptake not cancer-specific (improved with SPECT/CT), false-positive flare phenomenon, low spatial resolution
MRI	PET/MRI	No	Morphologic	Signal contrast depends on proton density (water content)	Sensitive for tumor within marrow, high spatial and contrast resolution, no radiation	Insensitive for mineralized bone, relatively expensive
		No	DW-MRI	Signal contrast depends on restriction of water molecule motion.	High sensitivity for tumor, no contrast required, quantitative changes in signal with therapy	May be less sensitive for sclerotic lesions, longer scan acquisition time
		No	DCE-MRI	Signal contrast depends on blood flow and perfusion.	Quantitative changes in signal with therapy	Requires IV contrast, requires modeling for parameter measurement
PET	PET/CT, PET/MRI	Yes	18F-fluoride	Uptake depends on blood flow and mineralization rate	Sensitive for detection, high contrast resolution, tomographic images in any plane	Uptake not cancer-specific (improved with PET/CT), false-positive flare phenomenon, relatively expensive
		Yes	18F-FDG	Uptake depends on tumor glucose transporters and glycolytic metabolism.	Tomographic images in any plane, sensitive for detection and therapy response assessment	Less sensitive for osteoblastic metastases and prostate cancer, relatively expensive
		Yes	11C/18F-choline	Uptake depends on choline transporters and choline kinase activity (cell membrane turnover).	Tomographic images in any plane, good sensitivity in prostate cancer	Insensitive at low PSA levels (eg, <1 ng/mL), relatively expensive
		Yes	68Ga-PSMA	Uptake depends on the level of tumor PSMA expression	Tomographic images in any plane, high sensitivity and specificity for prostate cancer in bone and soft tissues	Not specific to prostate cancer, relatively expensive

CT = computed tomography; SPECT = single photon emission computed tomography; MDP = methylene diphosphonate; PET = positron emission tomography; FDG = fluorodeoxyglucose; PSMA = prostate-specific membrane antigen; DW-MRI = diffusion-weighted magnetic resonance imaging; DCE-MRI = dynamic contrast-enhanced MRI; IV = intravenous; 11C = carbon-11; 18F = fluorine-18; 68Ga-gallium.
